# Blood Safety: The Madhya Pradesh Centralized Nucleic Acid Testing (NAT) Model for Blood Donor Screening

**DOI:** 10.7759/cureus.100798

**Published:** 2026-01-05

**Authors:** Ashok Yadav, Ruby Khan, Amrita Tripathi, Rahul Singh Bhadauria, Puneet Tandon, Rajni Choudhary

**Affiliations:** 1 Immuno-Hematology and Blood Transfusion, Mahatma Gandhi Memorial Medical College, Indore, IND; 2 Pathology, National Health Mission, Bhopal, IND; 3 Hematology, National Health Mission, Bhopal, IND; 4 Pathology, Gandhi Medical College, Bhopal, Bhopal, IND

**Keywords:** blood transfusion, centralized nat, hbv, hcv, hiv, madhya pradesh, nucleic acid testing, transfusion-transmitted infections

## Abstract

Background: Ensuring safe blood transfusion is a public health priority in India, where reliance on serological screening alone may miss early infections. Centralized nucleic acid testing (NAT) offers an opportunity to strengthen blood safety through advanced molecular diagnostics.

Aim: This study aimed to highlight the successful implementation of the Madhya Pradesh centralized NAT hub and spoke model for enhancing blood transfusion safety.

Methods: The current project was implemented through a public-private partnership (PPP) using a hub-and-spoke model. To assess its merit, a retrospective analysis of program data was conducted between January 2023 and September 2024 across Madhya Pradesh, India. Deidentified records from 24 satellite blood centers, including district hospitals and specialized blood banks, were processed at central hubs in Indore and Bhopal, which used the Roche cobas® TaqScreen MPX test (version 2.0). Operational challenges and performance indicators were reviewed.

Results: A total of 158,493 seronegative samples were processed, of which 943 (1 in 168) were NAT-reactive and would have been missed by routine serological screening, potentially preventing 2829 transfusion-transmitted infections. The program maintained a 48-hour turnaround time from sample collection to reporting. Operational challenges, such as standardization of sample handling, maintenance of the cold chain during transport, and high testing volumes, were successfully addressed through rigorous protocols and training.

Conclusion: The Madhya Pradesh centralized NAT model is feasible, effective, and efficient, demonstrating its potential to reform blood safety in India. This model may serve as a replicable framework for other developing countries facing similar challenges in transfusion medicine.

## Introduction

Blood donation is crucial to healthcare, with approximately 120 million units donated worldwide each year. India relies on over 4000 regulated blood banks to meet its continuous demand [1]. Madhya Pradesh, home to over 72 million people [2,3], currently has 174 blood centers, including 70 government-run facilities [4]. Earlier assessments reported 133 functional blood banks, with nearly half publicly owned and the rest managed by private or not-for-profit sectors [5,6]. Voluntary donations account for 91.1% of the state’s blood supply, while replacement donations constitute 8.9% [7]. Transfusion-transmitted infections (TTIs), including human immunodeficiency virus (HIV), hepatitis B virus (HBV), hepatitis C virus (HCV), syphilis, and malaria, pose a significant risk, with 1-2 infections per 1000 transfusions and high morbidity or mortality when transfusions are delayed [7-9]. In India, transfusion-transmitted infections (TTIs) such as HIV, HBV, HCV, syphilis, and malaria demonstrate prevalence rates of 1.6%-2.2% [8], and studies from Madhya Pradesh have reported similar trends, with HBV, HCV, and HIV prevalence between 2011-2017 and 2020-2022 remaining consistent at approximately 1.14%-1.34%, 0.10%-0.11%, and 0.08%-0.10%, respectively [10-12].

Although serological testing is mandatory for blood screening, its limitations, particularly in detecting early-stage infections, highlight the need for advanced methods such as nucleic acid testing (NAT) [9,13,14]. NAT utilizes polymerase chain reaction (PCR)-based amplification of viral ribonucleic acid or deoxyribonucleic acid for the real-time detection of HIV, HBV, and HCV, enabling significantly earlier identification of infections compared to antibody- or antigen-based tests [15,16]. In standard workflows, seroreactive samples are discarded, while seronegative donations undergo NAT, and NAT-reactive units are also excluded [17]. NAT substantially shortens the diagnostic window period, identifies occult infections, and improves transfusion safety [9,17]. The World Health Organization (WHO) recommends screening for HIV, HBV, HCV, and syphilis using serology and NAT, and many countries have adopted centralized hub-and-spoke NAT models to enhance efficiency and safety [18]. Despite cost and technical barriers to widespread adoption in India [16], NAT is considered cost-effective in high-prevalence settings because early detection prevents the downstream burden of treating window-period infections, particularly for HBV and HCV [19]. Centralized testing improves coordination, ensures standardized practices, optimizes resources, and expands access to high-quality blood screening.

The study evaluated the feasibility and effectiveness of the centralized NAT program implemented for blood donor screening in Madhya Pradesh. It estimated the NAT yield for HIV, HBV, and HCV among seronegative donations, assessed site-wise and regional variations in yield, and quantified the program’s potential impact on reducing transfusion-transmitted infections. The objective was to demonstrate the successful implementation of the state’s centralized NAT hub-and-spoke model and to provide a comprehensive analysis of NAT detection rates across multiple donor sites.

## Materials and methods

Study design

The current project was implemented through a public-private partnership (PPP) using a hub-and-spoke model. To assess its merit, a retrospective analysis of program data was conducted between January 2023 and September 2024 across Madhya Pradesh, India. Deidentified records from 24 satellite blood centers, including district hospitals and specialized blood banks, were processed at central hubs in Indore and Bhopal.

This study was noninterventional in nature and utilized deidentified clinical data collected as part of routine care from satellite blood centers across the state of Madhya Pradesh, India. The study relied solely on existing, deidentified records and did not involve any direct patient contact, interventional procedures, or patient participation; therefore, institutional ethics committee clearance/approval and individual informed consent were not required. All data were managed in compliance with standard confidentiality safeguards to ensure privacy and data security.

Setting

The healthcare setting of Madhya Pradesh includes a mix of government district hospitals and specialized blood banks. Blood donation practices in the state follow national guidelines, with serological testing being the conventional method for screening donations. The hub-and-spoke model allowed centralized testing, reducing the burden on individual collection sites while ensuring higher sensitivity in blood screening. NAT screening was used to improve the detection of HIV, HBV, and HCV in donated blood samples.

Adaptation of NAT through PPP

**Figure 1:**
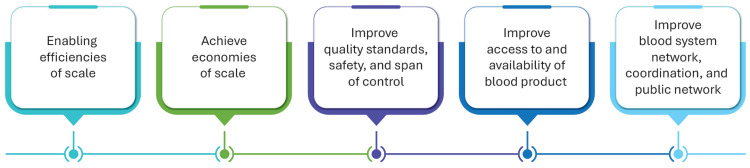
Benefits of centralization testing/public-private partnership model The image is created by the author.

The PPP model addressed human resource requirements, infrastructure, expertise development, and the smooth day-to-day operations of NAT. The PPP model was chosen to benefit from the private sector's expertise in laboratory management and technology while ensuring state oversight and monitoring (Figure 1).

A phased implementation was adopted, beginning with the establishment of NAT PCR laboratories at the Department of Transfusion Medicine (central hubs) in Mahatma Gandhi Memorial Medical College (MGM), Indore, and Gandhi Medical College (GMC), Bhopal. Satellite blood centers supported the centralized testing facilities across 24 district hospitals and specialized blood banks in the state. The private partner provided infrastructure development expertise, set up the laboratories, and trained laboratory personnel to ensure the successful implementation of NAT. Logistics support was facilitated by a distributor channel partner who handled sample pickups from district hospitals and delivered them to the hub sites. The State Blood Transfusion Council served as the nodal body, overseeing and supervising the process through designated nodal officers at both central hubs.

Training of personnel

Before the implementation of the model, all personnel involved in blood collection, processing, and transport at both satellite and hub centers underwent training. This included the proper labeling and handling of samples, the use of cold chain storage for sample transport, and the protocols for NAT PCR testing. Staff at the central hubs received training in operating the cobas® TaqScreen MPX Test, version 2.0 (Roche Diagnostics, Mannheim, Germany), a highly sensitive NAT assay that detects HIV, HBV, and HCV, and data management practices to ensure smooth workflow and maintenance of high-quality standards throughout the study.

Implementation process

**Figure 2:**
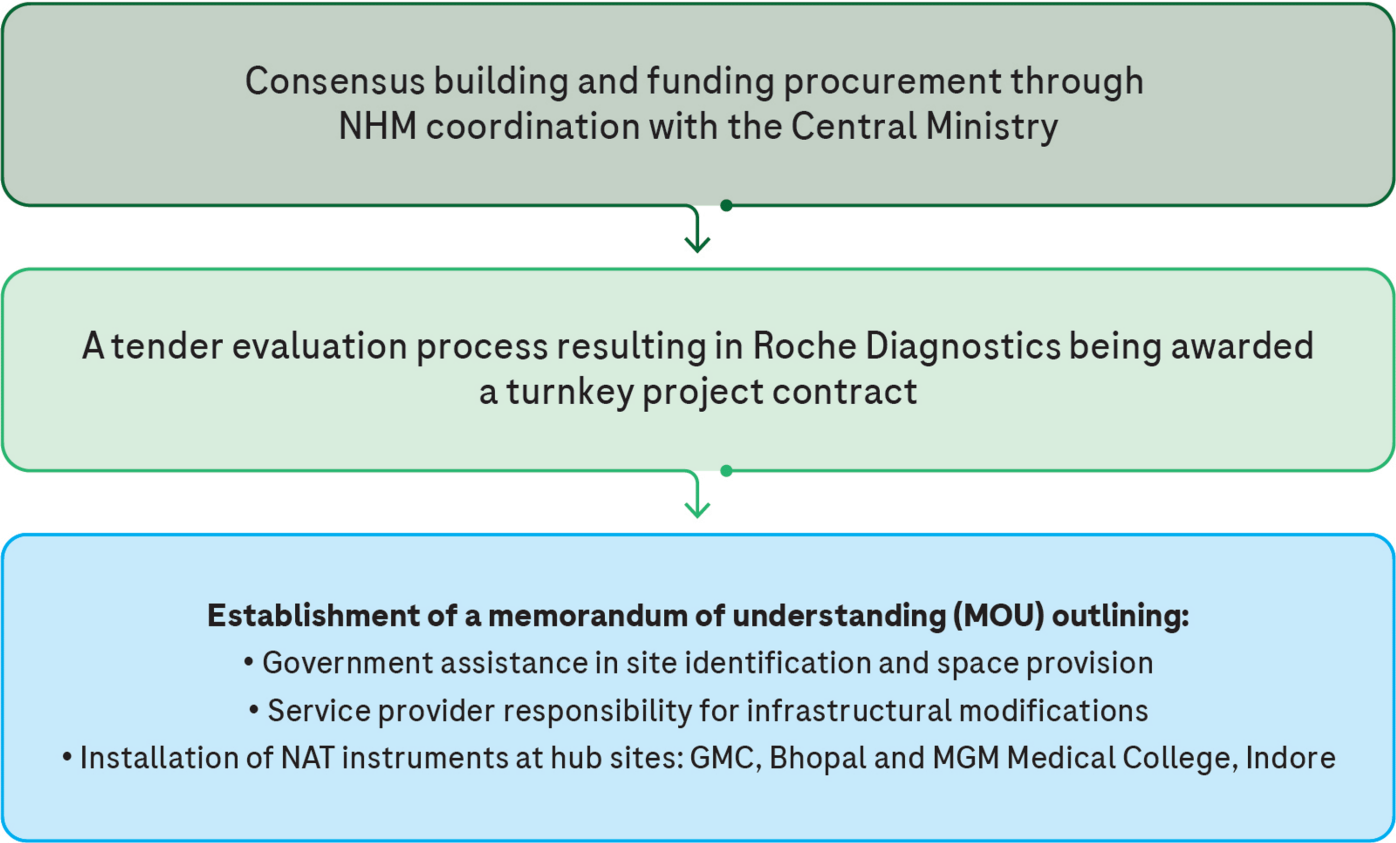
NAT PCR implementation process GMC: Gandhi Medical College; MGM: Mahatma Gandhi Memorial; MOU: Memorandum of Understanding; NAT: Nucleic acid test; NHM: National Health Mission. The image is created by the author.

Prior to the implementation of the model, a Technical Research Group was established, comprising officials from the National Health Mission, key government stakeholders, and transfusion medicine specialists in Madhya Pradesh. The implementation process is illustrated in Figure 2.

**Figure 3:**
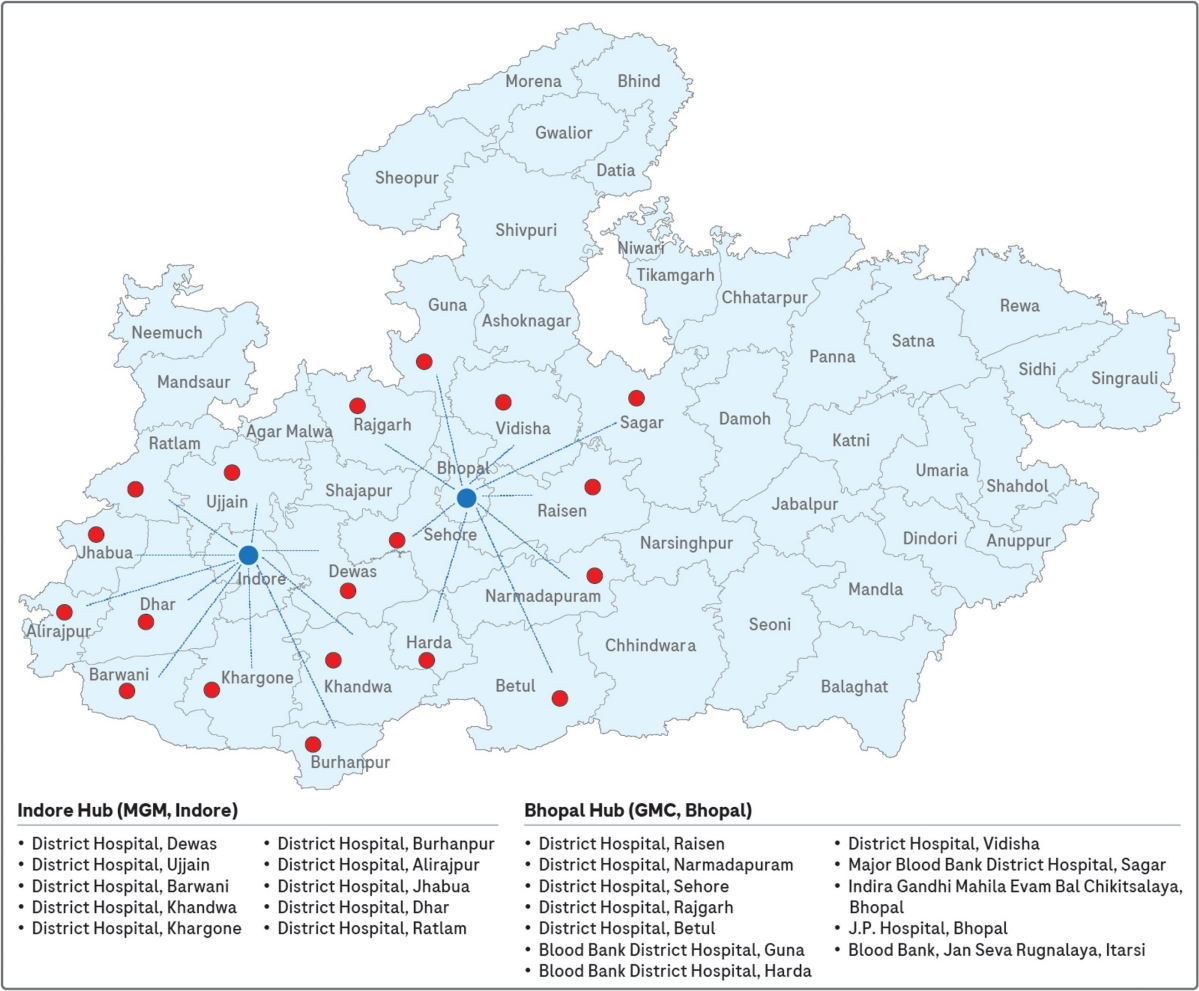
Hub-and-spoke model Disclaimer: The map of Madhya Pradesh used in this figure is for illustrative purposes only and may not accurately represent the current boundaries. No political or geographical assertions are intended. Please refer to official sources for accurate and up-to-date information. The image is created by the author.

For the first year, NAT was conducted at the hub sites to assess the efficacy and viability of the testing process. In 2022, the NAT facility was expanded to include 20 additional district hospitals under the hub-and-spoke model. The central hub in Indore was based at MGM Indore, and it catered to satellite blood centers in the surrounding region, including district hospitals in Dewas, Ujjain, Barwani, Khandwa, Khargone, Burhanpur, Alirajpur, Jhabua, Dhar, and Ratlam. Similarly, the central hub in Bhopal, located at GMC Bhopal, included the district hospitals of Raisen, Hoshangabad, Sehore, Rajgarh, Betul, Guna, Harda, Vidisha, and the Major Blood Bank at District Hospital Sagar. In addition, samples from Indira Gandhi Mahila Evam Bal Chikitsalaya and J.P. Hospital in Bhopal and the Blood Bank at Jan Seva Rugnalaya, Itarsi, were also processed at the Bhopal hub. Figure 3 illustrates the hub-and-spoke model implemented in this study.

NAT screening procedure

**Figure 4:**
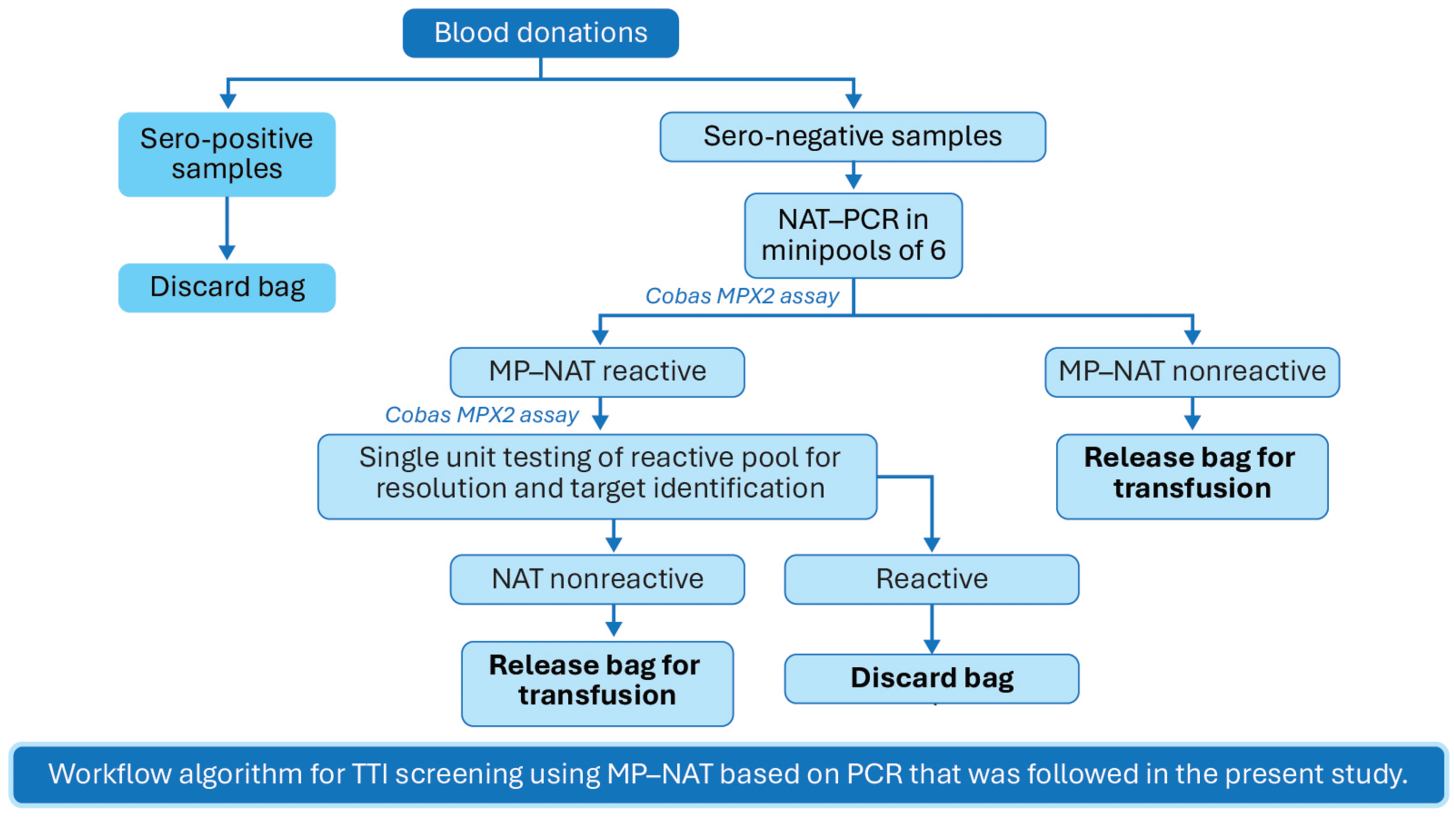
Workflow algorithm for TTI screening using MP-NAT based on PCR that was followed in the present study MP: Minipool; NAT: Nucleic acid testing; PCR: Polymerase chain reaction; TTI: Transfusion-transmitted infections. The image is created by the author.

**Figure 5:**
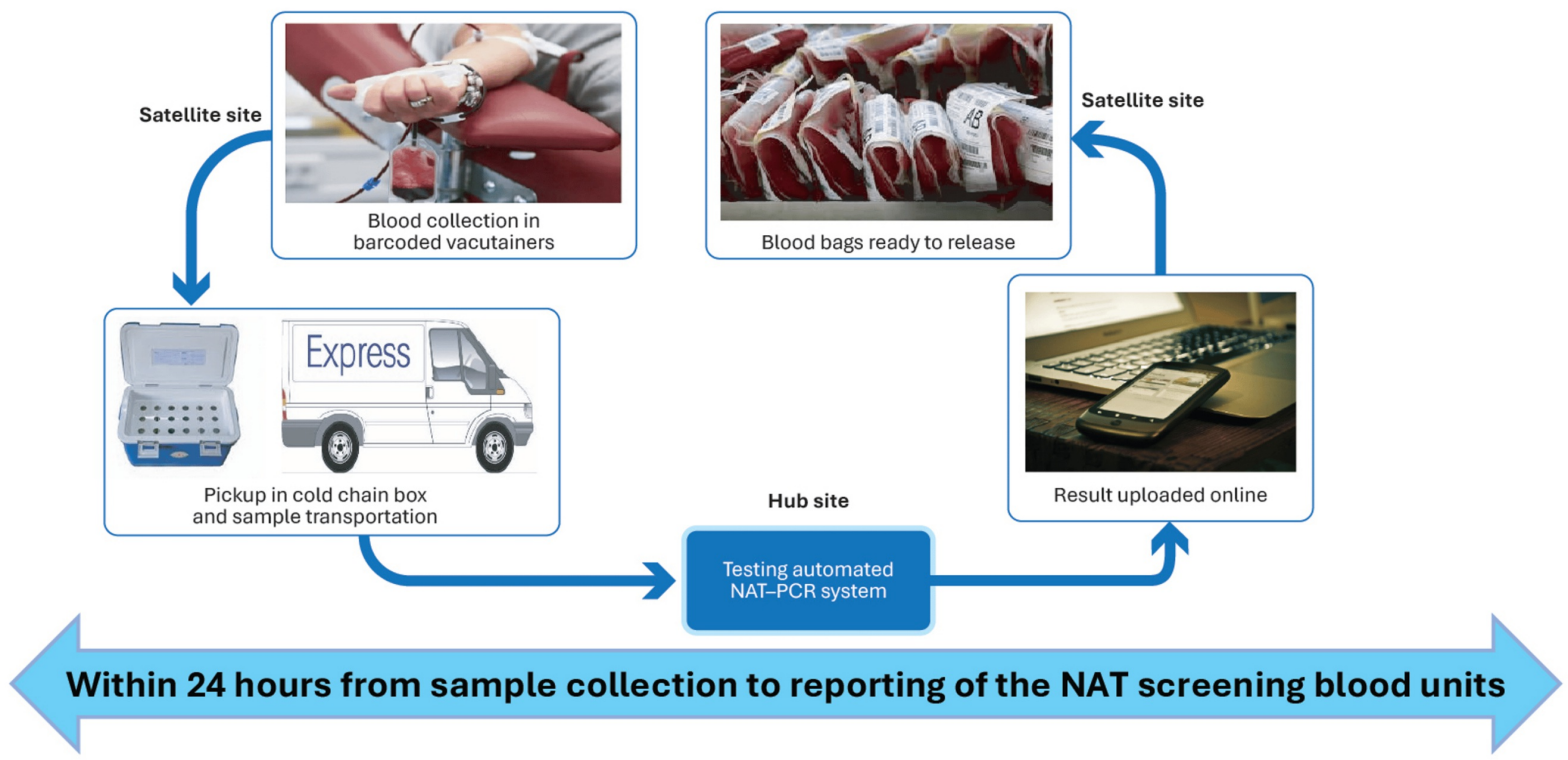
NAT centralized testing model workflow NAT: Nucleic acid testing; PCR: Polymerase chain reaction. The image is created by the author.

All serononreactive blood donations from the satellite blood centers were screened in six pools using the Roche cobas® TaqScreen MPX test, version 2.0 (MPX2), in a Minipool NAT (MP-NAT) format. Screening was conducted in fully automated NAT PCR systems at the central laboratories. The centralized NAT screening workflow (Figures 4, 5) began with blood collection in barcoded ethylenediaminetetraacetic acid tubes at the satellite centers, followed by plasma separation through gentle mixing and on-site centrifugation.

The separated plasma samples were then securely transported to the central testing hubs in Indore and Bhopal under cold chain conditions, maintaining a temperature of 2°C-8°C and reaching the hubs within 24 hours of collection. At the central laboratories, samples were processed using the fully automated Roche MP6 system with the cobas® MPX assay for amplification and detection. Test results were managed through a centralized data management system and uploaded online, allowing satellite centers to access results within 24 hours of collection. This workflow ensured sample integrity, efficient MP-NAT screening, and timely communication of results.

Data collection

For the analysis, deidentified data on donor demographics, infection rates, and testing outcomes were collected from each satellite blood center. Donor information, including age, gender, donation history, and geographical location, was recorded at the time of donation. All blood samples underwent initial serological screening for HIV, HBV, and HCV, and only serononreactive donations were forwarded for NAT PCR testing using the cobas® TaqScreen MPX test (version 2.0). NAT results were categorized as either nonreactive or reactive for HIV, HBV, or HCV, and NAT-reactive samples were flagged for further review. Using unique hospital codes, a centralized data management system was employed to track samples, manage results, and ensure confidentiality.

NAT yield calculation

The NAT yield was defined as the number of previously serononreactive blood samples that tested positive for HIV, HBV, or HCV through NAT PCR. The yield reflected the cases missed by conventional serological testing but detected by NAT. The overall NAT yield for each site and virus type was expressed as the number of samples tested per one NAT-reactive case.

Impact calculation

This study calculated the impact of NAT by tripling the number of reactive samples (NAT yield). The impact was calculated as estimated lives saved = NAT yield × 3. This multiplication accounts for the fact that each blood donation is typically separated into three components (red blood cells, plasma, and platelets) before transfusion. Each component can be transfused to different recipients, meaning that a single infected blood sample can potentially transmit infection to three individuals.

Statistical analysis

Descriptive statistics summarized the data, including the total number of samples tested, the number of reactive samples, and the calculated NAT yield per virus. All statistical analyses and graphical representations, including cumulative yield plots and comparative charts, were conducted and generated using an Excel spreadsheet (Microsoft Excel 365, Microsoft Corp., USA).

## Results

Sample testing overview

A total of 158,493 seronegative blood samples were collected from various donor sites in Madhya Pradesh between 2023 and 2024. Of the total samples, 943 tested reactive through NAT, yielding an overall NAT reactivity rate of 1 in 168.

District-wise NAT yield

The NAT yield varied across different donor sites. The highest yield was observed at District Hospital, Hoshangabad, with a NAT yield of 1 in 57, while District Hospital, Barwani, and District Hospital, Ratlam, reported no NAT reactive samples. Site-specific yield details for HIV, HBV, and HCV are presented in Table 1.

**Table 1:** NAT yield by donor site GMC: Gandhi Medical College; MGM: Mahatma Gandhi Memorial; HBV: Hepatitis B virus; HCV: Hepatitis C virus; HIV: Human immunodeficiency virus; NAT: Nucleic acid testing.

Site	Samples tested	Total NAT reactive	HIV reactive	HBV reactive	HCV reactive	NAT yield (total)	HIV yield	HBV yield	HCV yield
Total	158,493	943	53	815	69	1 in 168	1 in 2990	1 in 194	1 in 2297
District Hospital, Dewas	2689	33	1	32	0	1 in 81	1 in 2689	1 in 84	0
District Hospital, Ujjain	5327	7	1	5	1	1 in 761	1 in 5327	1 in 1065	1 in 5327
District Hospital, Barwani	45	0	0	0	0	0	0	0	0
District Hospital, Khandwa	247	4	0	4	0	1 in 62	0	1 in 62	0
District Hospital, Khargone	4706	23	0	23	0	1 in 205	0	1 in 205	0
District Hospital, Burhanpur	677	5	1	4	0	1 in 135	1 in 677	1 in 169	0
District Hospital, Alirajpur	233	1	0	1	0	1 in 233	0	1 in 233	0
District Hospital, Jhabua	1764	12	0	9	3	1 in 147	0	1 in 196	1 in 588
GMC, Bhopal	26,391	99	9	79	12	1 in 267	1 in 2932	1 in 334	1 in 2199
MGM, Indore	60,295	336	23	291	22	1 in 179	1 in 2622	1 in 207	1 in 2741
District Hospital, Raisen	1237	19	0	19	0	1 in 65	0	1 in 65	0
District Hospital, Hoshangabad	4539	80	9	71	2	1 in 57	1 in 504	1 in 64	1 in 2270
District Hospital, Sehore	7898	30	2	27	1	1 in 263	1 in 3949	1 in 293	1 in 7898
District Hospital, Rajgarh	6252	17	1	17	0	1 in 368	1 in 6252	1 in 368	0
District Hospital, Dhar	3972	18	0	18	0	1 in 221	0	1 in 221	0
District Hospital, Betul	3108	16	3	13	0	1 in 194	1 in 1036	1 in 239	0
Major Blood Bank District Hospital, Sagar	13818	119	11	98	10	1 in 116	1 in 1256	1 in 141	1 in 1382
Blood Bank District Hospital, Guna	2808	9	0	7	2	1 in 312	0	1 in 401	1 in 1404
Blood Bank District Hospital, Harda	1983	24	3	21	0	1 in 83	1 in 661	1 in 94	0
District Hospital, Vidisha	5785	82	8	61	14	1 in 71	1 in 723	1 in 95	1 in 413
Indira Gandhi Mahila Evam Bal Chikitsalaya, Bhopal	880	2	0	2	0	1 in 440	0	1 in 440	0
J. P. Hospital, Bhopal	1737	4	0	2	2	1 in 434	0	1 in 869	1 in 869
Blood Bank, Jan Seva Rugnalaya, Itarsi	1732	3	3	9	0	1 in 577	1 in 577	1 in 192	0
District Hospital, Ratlam	370	0	0	2	0	0	0	1 in 185	0

NAT yield by virus

NAT reactivity for HBV was the highest among the three infections, with 815 reactive samples, representing a yield of 1 in 194 across all sites. HIV was the least frequent, with only 53 reactive samples, corresponding to a yield of 1 in 2990. HCV was detected in 69 samples, with a yield of 1 in 2297. The breakdown of the yield for each virus type per site is shown in Table 1. Additionally, sites such as District Hospital, Hoshangabad, and Major Blood Bank, Sagar, reported relatively higher yields across all three viruses. In contrast, smaller sites like District Hospital, Barwani, and District Hospital, Ratlam, had either no reactive samples or minimal detection.

Cumulative yield for 2023-2024

Over the two years from 2023 to 2024, NAT yield rates remained relatively stable across the sites, with a cumulative yield of 1 in 168. The detection of HBV remained the most consistent, while HCV and HIV yields showed more sporadic occurrences.

Impact of NAT on lives saved

The implementation of NAT in blood screening has the potential to save numerous lives. In total, 943 samples tested reactive through NAT, translating to an estimated 2829 lives saved across all infections, as each yield potentially saves three lives due to the separation of each blood bag into three components before transfusion. When analyzing the impact of NAT on HBV, the testing identified 815 reactive samples, leading to an estimated 2445 lives saved. The NAT results for HIV showed 53 reactive samples, resulting in approximately 159 lives saved. Lastly, there were 69 reactive samples for HCV, translating to an estimated 207 lives saved.

## Discussion

The implementation of a centralized NAT hub-and-spoke model across blood banks in Madhya Pradesh demonstrated a substantial impact on blood safety. The study processed 158,493 seronegative blood samples from 24 satellite blood centers, including district hospitals and blood banks, through two central hubs in Indore and Bhopal. The overall NAT reactivity rate was 1 in 168 samples, with HBV showing the highest yield at 1 in 194, compared to HIV (1 in 2990) and HCV (1 in 2297). These findings underscore the ability of NAT to detect infections that conventional serological testing may miss, including occult hepatitis B infections (OBI) and window-period infections [20,21]. The centralized NAT approach potentially prevented the transfusion of 943 infected blood donations, resulting in an estimated 2829 lives saved, considering that each donation is typically separated into three components (red blood cells, plasma, and platelets) [22].

Significant variations in NAT yields were observed across different sites; for instance, District Hospital, Hoshangabad, had a high yield of 1 in 57, whereas District Hospital, Barwani, reported no reactive samples. These differences may reflect local prevalence rates, demographic factors, or variations in predonation screening practices, highlighting the importance of targeted interventions and resource allocation based on local epidemiology. The Madhya Pradesh model provides a feasible blueprint for enhancing blood safety nationwide, incorporating centralized hubs, standardized procedures, efficient logistics, and personnel training. Other states could adopt similar phased rollouts, beginning with central hubs and expanding to satellite centers, creating a scalable and efficient blood safety system [23,24].

Centralized NAT facilities offer multiple advantages, including enhanced detection accuracy, reduced turnaround time, minimized workflow redundancy, and improved operational control. Smaller, resource-limited blood banks can benefit from access to advanced testing technologies without incurring significant investments, thereby reducing urban-rural disparities and improving blood safety [18,25,26]. The use of the cobas® TaqScreen MPX test (version 2.0) ensured standardized testing procedures across the state, critical for quality control and reliable data comparison. High clinical specificity, broader genotypic coverage, and automated, user-friendly features contributed to the system's efficacy [21].

Traditional methods, such as rapid diagnostic tests and serological assays, though widely used, have limitations in detecting low viral loads or infections during the window period [27-30]. Chemiluminescent immunoassays, while more sensitive than conventional enzyme-linked immunosorbent assays and rapid diagnostic tests, remain costly, have limited test panels, and may still miss early-stage infections [26]. NAT, therefore, is vital in enhancing transfusion safety, particularly for detecting OBI infection and pre-seroconversion infections [20]. Evidence from India and globally supports the effectiveness of NAT; a review across 11 publications revealed NAT yields of 1 in 1761 for HBV, 1 in 5484 for HCV, and 1 in 66,000 for HIV, highlighting its critical role even where NAT is limited to a minority of blood banks [8,23].

The operational challenges of implementing centralized NAT, such as inconsistent sample labeling and temperature control during transport, were mitigated through the use of standardized barcoded tubes, insulated storage boxes with cold gel packs, and dedicated sample collection porters. Twice-daily testing ensured a 24-hour turnaround time, and a centralized data management system maintained donor confidentiality and efficient sample tracking. Blood units that were nonreactive were promptly cleared for clinical use, ensuring timely availability for patients.

Blood component therapy adds additional urgency to early detection, as one infected unit can affect multiple recipients. In Madhya Pradesh, approximately 67.9% of collected blood units with component separation facilities undergo separation into red blood cells, plasma, and platelets [6,31,32]. NAT's role in detecting low viral load infections is thus particularly relevant in preventing the spread of transfusion-transmitted infections. Previous studies demonstrated that detecting 24 HBV carriers through NAT potentially prevented 75 patients from receiving infected blood components [22].

While NAT implementation requires investment, the cost is offset by the lives saved and the improved safety of blood supplies. The Madhya Pradesh model demonstrates that centralized NAT, with its logistical and operational efficiencies, provides a replicable framework for other Indian states. Limitations of this study include its confinement to Madhya Pradesh, which may limit generalizability due to regional variations in TTI prevalence. Additionally, cost-effectiveness analyses were not included, which could be valuable for policy decisions. Future research should expand centralized NAT evaluation to other states, providing a comprehensive understanding of its impact on national blood safety.

Limitations

This retrospective, programmatic analysis did not include donor follow-up or independent evaluation of assay-level quality-control metrics. The estimated impact of NAT is based on assumptions regarding routine blood component separation and should be interpreted as a theoretical projection. Additionally, the findings are derived from a single state and may not be generalizable to settings with different epidemiological or operational characteristics.

## Conclusions

Implementing a centralized hub-and-spoke model for NAT screening in Madhya Pradesh has proven to be logistically feasible and operationally efficient, particularly in regions where smaller blood banks lack advanced testing infrastructure. Given the high burden of TTIs in India, the adoption of NAT has the potential to reduce transfusion-related infectious risk by enabling earlier detection of HIV, HBV, and HCV. PCR-based NAT provides high sensitivity, specificity, and broad viral coverage and was effective in identifying previously undiagnosed infections among seronegative donations in this program. These findings support the role of centralized NAT in enhancing blood safety and offer programmatic insights for policymakers and healthcare administrators in India and other resource-constrained settings considering similar models.
